# Streamlined alpha-synuclein RT-QuIC assay for various biospecimens in Parkinson’s disease and dementia with Lewy bodies

**DOI:** 10.1186/s40478-021-01175-w

**Published:** 2021-04-07

**Authors:** Connor Bargar, Wen Wang, Steven A. Gunzler, Alexandra LeFevre, Zerui Wang, Alan J. Lerner, Neena Singh, Curtis Tatsuoka, Brian Appleby, Xiongwei Zhu, Rong Xu, Vahram Haroutunian, Wen-Quan Zou, Jiyan Ma, Shu G. Chen

**Affiliations:** 1 Department of Pathology, Case Western Reserve University School of Medicine, Cleveland, OH 44106 USA; 2 Department of Neurology, University Hospitals Cleveland Medical Center, Case Western Reserve University School of Medicine, Cleveland, OH 44106 USA; 3 University of Maryland Brain and Tissue Bank, Baltimore, MD 21201 USA; 4 Department of Population and Quantitative Health Sciences, Case Western Reserve University School of Medicine, Cleveland, OH 44106 USA; 5 Department of Psychiatry, Icahn School of Medicine at Mount Sinai, New York, NY 10029 USA; 6 Van Andel Institute, Grand Rapids, MI 49503 USA

**Keywords:** Alpha-synuclein, Biomarker, Biospecimens, Cerebrospinal fluid, Colon, Dementia with Lewy bodies, Parkinson’s disease, RT-QuIC, Salivary gland, Skin

## Abstract

Definitive diagnosis of Parkinson’s disease (PD) and dementia with Lewy bodies (DLB) relies on postmortem finding of disease-associated alpha-synuclein (αSyn^D^) as misfolded protein aggregates in the central nervous system (CNS). The recent development of the real-time quaking induced conversion (RT-QuIC) assay for ultrasensitive detection of αSyn^D^ aggregates has revitalized the diagnostic values of clinically accessible biospecimens, including cerebrospinal fluid (CSF) and peripheral tissues. However, the current αSyn RT-QuIC assay platforms vary widely and are thus challenging to implement and standardize the measurements of αSyn^D^ across a wide range of biospecimens and in different laboratories. We have streamlined αSyn RT-QuIC assay based on a second generation assay platform that was assembled entirely with commercial reagents. The streamlined RT-QuIC method consisted of a simplified protocol requiring minimal hands-on time, and allowing for a uniform analysis of αSyn^D^ in different types of biospecimens from PD and DLB. Ultrasensitive and specific RT-QuIC detection of αSyn^D^ aggregates was achieved in million-fold diluted brain homogenates and in nanoliters of CSF from PD and DLB cases but not from controls. Comparative analysis revealed higher seeding activity of αSyn^D^ in DLB than PD in both brain homogenates and CSF. Our assay was further validated with CSF samples of 214 neuropathologically confirmed cases from tissue repositories (88 PD, 58 DLB, and 68 controls), yielding a sensitivity of 98% and a specificity of 100%. Finally, a single RT-QuIC assay protocol was employed uniformly to detect seeding activity of αSyn^D^ in PD samples across different types of tissues including the brain, skin, salivary gland, and colon. We anticipate that our streamlined protocol will enable interested laboratories to easily and rapidly implement the αSyn RT-QuIC assay for various clinical specimens from PD and DLB. The utilization of commercial products for all assay components will improve the robustness and standardization of the RT-QuIC assay for diagnostic applications across different sites. Due to ultralow sample consumption, the ultrasensitive RT-QuIC assay will facilitate efficient use and sharing of scarce resources of biospecimens. Our streamlined RT-QuIC assay is suitable to track the distribution of αSyn^D^ in CNS and peripheral tissues of affected patients. The ongoing evaluation of RT-QuIC assay of αSyn^D^ as a potential biomarker for PD and DLB in clinically accessible biospecimens has broad implications for understanding disease pathogenesis, improving early and differential diagnosis, and monitoring therapeutic efficacies in clinical trials.

## Introduction

An unmet clinical need is a reliable premortem diagnostic biomarker for Parkinson’s disease (PD) and dementia with Lewy bodies (DLB), two common synucleinopathies [1]. Currently, definitive diagnosis of PD and DLB relies mainly on postmortem detection of disease-associated alpha-synuclein (αSyn^D^), deposited as protein aggregates within the Lewy body inclusions in the central nervous system (CNS) [2, 3]. Overwhelming evidence suggests that αSyn^D^ aggregates are formed as a consequence of protein misfolding cascades and may spread through prion-like transmission across cells and tissues during disease pathogenesis [4]. These αSyn^D^ aggregates may potentially serve as a promising biomarker of PD and DLB for premortem diagnosis and treatment. However, detection of αSyn^D^ aggregates in easily accessible specimens has been difficult and a clinically validated assay platform remains to be established. Previously, immunoassays have been used to measure various forms of αSyn (total and phosphorylated) in cerebrospinal fluid (CSF), a CNS-derived body fluid. However, the conventional immunoassays of CSF in PD and DLB have thus far yielded inconsistent and disappointing performance for diagnostic purpose [5–7]. Similarly, immunohistochemical (IHC) staining of phosphorylated αSyn has been observed in peripheral tissues of PD and DLB, but wide variations in analytic performance have been reported by various groups as standardization of IHC staining remains a formidable challenge [8–12].

The real-time quaking induced conversion (RT-QuIC) assay has recently emerged as a powerful platform for amplified detection of αSyn^D^ aggregates [13]. The αSyn RT-QuIC assay monitors the template-seeded aggregation of recombinant monomeric αSyn into amyloid fibrils upon seeding by traces of αSyn^D^ aggregates present in biospecimens. The newly generated β-sheet rich amyloid fibrils bind to an amyloid-sensitive dye, thioflavin T (ThT), resulting an enhanced fluorescence. Recent studies have utilized αSyn RT-QuIC assay to detect seeding activity of αSyn^D^ aggregates in brain homogenate (BH) and CSF samples from PD and DLB, with a diagnostic sensitivity great than 90% and specificity of 82–100% for CSF samples [14–17]. Moreover, the αSyn RT-QuIC method has been extended to evaluate the presence of αSyn^D^ from non-CNS sources, such as nasal brushing [18], salivary gland [19] and skin [20, 21]. The RT-QuIC platform has the advantage of high throughput, fast turnaround, and automated recording of aggregation kinetics in real time. However, the published assay conditions for αSyn RT-QuIC vary in both configuration and the sources and compositions of reagents, thus discouraging a broad use of this novel technology in clinical research and disease diagnosis. For example, recombinant αSyn (rec-Syn), the RT-QuIC substrate, was often purified in-house from the expression in *E. coli*, either as the wild-type rec-Syn or an engineered mutant (rec-Syn-K23Q) [15]. This approach demands a considerable technical expertise and a lengthy hands-on time of ~ 5–7 days. A suitable commercial source of rec-Syn will greatly simplify the implementation of αSyn RT-QuIC assay in a routine laboratory. Moreover, existing RT-QuIC protocols also vary depending on the types of tissue specimens such as brain, CSF, salivary gland, and skin [15, 19–22]. However, it has been unclear whether different assay conditions are indeed required for different tissue matrices. Conceivably, a streamlined RT-QuIC protocol suitable for the detection of αSyn^D^ in a diverse array of biospecimens will facilitate the standardized assessment of clinically accessible tissues for the diagnosis of PD and DLB.

In the present study, we have developed a streamlined αSyn RT-QuIC assay through the optimized use of entirely commercial reagents, enabling rapid assembly of assay components with minimal hands-on time. We have validated this streamlined αSyn RT-QuIC assay using a large collection of CSF from neuropathologically confirmed cases of PD, DLB, and non-synucleinopathy (NS) controls, yielding high analytical performance in terms of sensitivity and specificity. Moreover, we have shown that a single RT-QuIC protocol is feasible for ultrasensitive and rapid assay of αSyn^D^ in PD across different types of tissues including the brain, skin, salivary gland, and colon.

## Methods

### Human specimens

All human samples were received as de-identified postmortem cases originated from the respective sites and collected under the guidelines of local ethics committees. Unless otherwise specified, frozen brain tissue and CSF samples for the development of αSyn RT-QuIC assay were obtained from the NIH NeuroBioBank (NBB), a centralized network of biorepositories. Brain tissue samples included 3 cases each of PD and DLB with confirmed neuropathology and 3 cases of NS controls archived at NBB. A collection of 214 CSF samples from NBB included cases of PD (n = 88), DLB (n = 58) and NS controls (n = 68) including those from the neurologically normal (n = 23), amyotrophic lateral sclerosis (ALS, n = 9), multiple sclerosis (MS, n = 6), Alzheimer’s disease (AD, n = 7), Pick’s disease (n = 10), corticobasal degeneration (CBD, n = 4), and progressive supranuclear palsy (PSP, n = 9). The demographic data of CSF cases examined for diagnostic validation by RT-QuIC analyses are listed in Table 1. Multiple tissue panels available from the same cadavers included a case of neuropathologically confirmed PD (case number 6164) and a NS control affected by AD (case number 5687) with samples of the brain, scalp skin, and CSF obtained from NBB (used for Fig. 4a), a case of neuropathologically confirmed PD (case number 2054) and a NS control without any neurological disease (case number 2074) with samples of scalp skin, submandibular gland (SMG), sigmoid colon, and CSF obtained from the Banner Sun Health Research Institute (BSHRI, Sun City, Arizona, USA) (used for Fig. 4b), and a case of neuropathologically confirmed PD (case number 2052) and a NS control without any neurological disease (case number 2078) with samples of scalp skin, SMG, sigmoid colon obtained from BSHRI (used for Fig. 5).

**Table 1:** Demographic information for CSF cases and results of αSyn RT-QuIC assay

	PD	DLB	NS Controls							
			Total	Normal	ALS	MS	AD	Picks	CBD	PSP
No	88	58	68	23	9	6	7	10	4	9
Age, years (SD)	78.3 (8.1)	77.2 (8.8)	74.3 (10.9)	79.6(8.3)	72.2 (9.6)	66.3 (9.0)	70.9 (16.7)	68.5 (10.7)	74.5 (4.2)	79.1 (10.1)
Male, no. (%)	58 (66)	37 (64)	36 (53)	14 (61)	3 (33)	0 (0)	3 (43)	10 (100)	3 (75)	3 (33)
RT-QuIC assay										
ThT at 60 h, % (SE)	54.5 (2.2)	64.0 (2.5)	5.2 (0.2)	5.8 (0.4)	5.5 (0.4)	5.5 (0.4)	3.8 (0.4)	4.3 (0.5)	4.4 (0.8)	6.2 (1.6)
* P* value (compared to PD)^a^	NA	< 0.05	< 0.0001	< 0.0001	< 0.0001	< 0.0001	< 0.0001	< 0.0001	< 0.0001	0.0003
* P* value (compared to NS total controls)^a^	< 0.0001	< 0.0001	NA	> 0.99	> 0.99	> 0.99	> 0.99	> 0.99	> 0.99	> 0.99

NA, not applicable

^a^ *P* values determined by one way analysis of variance with Tukey’s post hoc test

### Preparation of human specimens

All human biospecimens were received on dry ice and were stored at -80 °C before sample preparation. Frozen brain samples were lysed in ice-cold lysis buffer [Dulbecco’s phosphate-buffered saline (PBS) supplemented with 1% Triton X-100, 150 mM NaCl, 5 mM EDTA, and Roche mini-cOmplete™ proteinase inhibitors (Roche Diagnostics, Indianapolis, IN, USA)], followed by homogenization in the presence of zirconia beads (1 mm) in a mini-Beadbeater-16 device (BioSpec Products, Bartlesville, OK, USA) for 5 cycles of 1 min beating and 3 min cooling at 4 °C. Following a brief centrifugation for 5 min at 500 × g, a 10% brain homogenate (w/v) was prepared and stored at − 80 °C until use. For all peripheral tissues, previous studies included a 4-h enzymatic digestion step during tissue extraction [20, 23]. This step was eliminated in our protocol without noticeable changes in extraction efficiency. Frozen samples of scalp skin were thawed, and washed in ice-cold PBS at least three times until no visible blood remained. The skin tissue was minced with a razor blade, followed by homogenization in lysis buffer in the presence of zirconia beads as above. A 10% skin homogenate (w/v) was prepared and stored at -80 °C until use. Frozen samples of SMG and sigmoid colon were prepared in the same manner as the skin, as described above. A 10% homogenate (w/v) of SMG or colon was prepared and stored at -80 °C until use. Frozen samples of CSF were thawed and used immediately before assay without further processing.

### Reagents for RT-QuIC assay

All reagents used for αSyn RT-QuIC assay were obtained from commercial sources. Sodium phosphate (0.5 M, pH 8.0) was from Boston BioProducts (Ashland, MA, USA), sodium chloride (5 M) was from Invitrogen (Carlsbad, CA, USA), thioflavin T (ThT) was from Sigma (St. Louis, MO, USA). HPLC-grade water from Thermo Fisher (Waltham, MA, USA) was used to prepare solutions. Aliquots of individual stock solutions were stored at −20 °C until use. Lyophilized recombinant human αSyn (rec-Syn) were purchased from rPeptide (Watkinsville, GA, USA) with catalog number S-1001–02 and lot numbers 082517AS (lot 1) and 111317AS (lot 2), and stored at −20 °C until use.

### RT-QuIC assay procedure and data analysis

For tissue samples, an initial tissue homogenate [10% (w/v), defined as 10^–1^ dilution] as prepared above was subjected to serial tenfold dilutions with sample diluent containing PBS supplemented with Gibco 1 × N2 supplement (Thermo Fisher, Waltham, MA, USA). RT-QuIC reactions were performed in Nunc black 96-well plates with optical flat bottom (Thermo Fisher, Waltham, MA, USA). Each well was preloaded with six 0.8 mm low-binding silica beads (OPS Diagnostics, Lebanon, NJ, USA). Lyophilized rec-Syn (rPeptide) was reconstituted in HPLC-grade water to 1 mg/ml and filtered through Amicon 100 kDa filters (Millipore, Burlington, MA, USA) by centrifugation for 10 min at 4 °C. To measure αSyn RT-QuIC seeding activity, 2 μl of diluted tissue homogenate was added to individual wells containing 98 μl of RT-QuIC reaction mixture composed of 40 mM NaPO4 (pH 8.0), 170 mM NaCl, 20 µM ThT, 0.1 mg/ml rec-Syn. The plates were sealed with Nunc clear sealing film (Thermo Fisher, Waltham, MA, USA) and incubated at 42 °C in a BMG FLUOstar Omega plate reader (BMG Labtech, Cary, NC, USA) with cycles of 1 min shaking (400 rpm, double orbital) and 1 min rest throughout the assay. ThT fluorescence (450 nm excitation and 480 nm emission; bottom read) was recorded every 45 min for 60 h. For CSF samples, 2 μl of neat CSF or CSF serially diluted with sample diluent was added to individual wells containing 98 μl of RT-QuIC reaction mixture supplemented with 0.0005% SDS. Four replicate reactions were made for each sample. The plate reader was set up with optic gain setting (~ 1900) that would give a negative control baseline of ThT fluorescence around 15,000 relative fluorescence units (rfu). The maximal fluorescence response of the plate reader was fixed at 260,000 rfu. Raw data with ThT fluorescence intensity in rfu were normalized to a percentage of the maximal fluorescence at 260,000 rfu (defined as 100%). Positive RT-QuIC reactivity of individual wells is defined as enhanced ThT fluorescence above a predefined threshold within 60 h. This threshold was calculated as the average background fluorescence plus 5 standard deviations, equal to ~ 30,000 rfu or ~ 11% of maximum ThT response in our experiments. For RT-QuIC analyses, data from four technical replicates were examined for a given biospecimen. A biospecimen was considered positive when at least two out of the four replicates displayed positive ThT reactivity above the threshold. For each positive sample, the average fluorescence intensity from the positive replicates was calculated and plotted against time. For each negative sample, the average fluorescence intensity from the negative replicates was calculated and plotted against time. The lag phase of RT-QuIC reactions was defined as the time (h) to reach the threshold as defined above. Protein aggregation rate (PAR) was calculated as the inverse of lag-phase (1/h). For Spearman-Kärber analyses of end-point dilution RT-QuIC experiments, tenfold serial dilutions of a biospecimen were prepared. Each diluted sample was used to seed RT-QuIC reactions in quadruplicate. ThT fluorescence from individual replicates for each dilution were plotted against time. The dilution series continued until RT-QuIC reactivity reached ≤ 1 out 4 replicates. The seeding dose giving ThT positivity in 50% of replicate wells (SD_50_) based on the Spearman-Kärber equations was calculated as described [24, 25].

### Statistical analysis

The statistical comparisons were performed using t-tests and one-way ANOVA for comparing two or more groups, respectively (GraphPad Prism Software Version 9.0.0). Data were expressed as means ± SD (standard deviation) or SE (standard error of the mean). A two-sided type I error level of 0.05 was adopted. The statistically significant differences were expressed as * *p* < 0.05, ** *p* < 0.01, *** *p* < 0.005, and **** *p* < 0.001 or p < 0.0001, as indicated. The number of biological replicates was expressed as “n”, and 4 technical replicates was used for each biological sample.

## Results

### Streamlined αSyn RT-QuIC assay

Our streamlined RT-QuIC assay protocol was developed based on the second-generation platform of αSyn RT-QuIC as described by Caughey and colleagues [15], with the following modifications. We acquired all assay reagents from commercial sources, including the monomeric rec-Syn protein, the substrate present in vast excess for in vitro conversion into amyloid fibrils by seeding with minute quantities of αSyn^D^ aggregates from a clinical sample to be examined. This approach eliminated the need for the laborious and lengthy (~ 5–7 days) in-house production and purification of rec-Syn by highly specialized personnel, and minimized the variability in the quality of rec-Syn produced [15]. Following a screen of rec-Syn products from several sources, we found that a commercial product of monomeric human wild-type rec-Syn (rPeptide) was most suitable for this RT-QuIC assay (see Methods section). Two separate lots of the commercial rec-Syn protein were successfully used for the RT-QuIC assay with ~ 300 reaction plates over a two-year period. Accordingly, our simplified αSyn RT-QuIC assay involved minimal hands-on time (~ 1 h) to rapidly assemble all reagents for RT-QuIC reactions in a 96-well plate, sufficient for testing 24 samples in quadruplicate. Moreover, we markedly shortened the preanalytical processing of peripheral tissues by eliminating a 4-h enzymatic digestion step used in previous studies [20, 23]. In addition, the ThT concentration was increased from 10 µM to 20 µM to minimize self-quenching of ThT fluorescence [26]. Finally, we found that a single streamlined RT-QuIC assay protocol worked equally well for several different types of tissues including the brain, skin, salivary gland, and colon.

### RT-QuIC assay of αSyn^D^ seeding activity in brain homogenates of PD and DLB

**Fig. 1:**
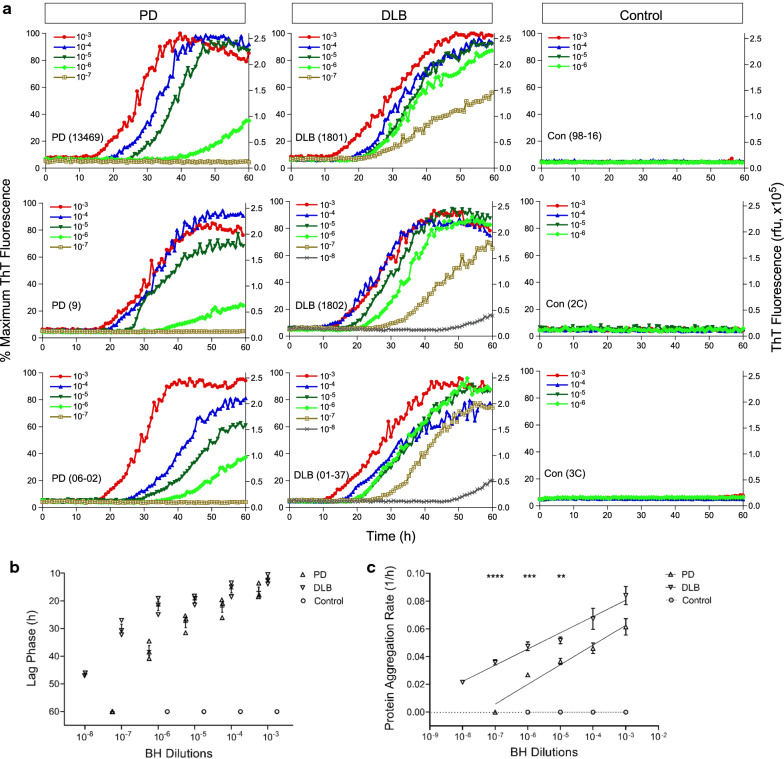
αSyn RT-QuIC analysis of PD and DLB brain samples. **a** αSyn RT-QuIC spectra of BH from neuropathologically confirmed cases of PD (n = 3, left panels), DLB (n = 3, middle panels), and NS controls (n = 3, right panels). Case numbers were indicated in parentheses. Two µl of BH diluted to 10^–3^ through 10^–8^ (w/v) was used for seeding RT-QuIC reactions in quadruplicate. Average RT-QuIC reactivity was shown for individual BH dilutions of each of 3 cases tested in quadruplicate. Data were expressed as percentages of the maximum ThT fluorescence (left Y-axis), with corresponding relative fluorescence units (rfu, right Y-axis). **b** Lag phase of RT-QuIC reactions from individual dilutions of PD, DLB, and control samples. RT-QuIC spectra for individual dilutions of each case in **a** were used to obtain the time (h) required for the average fluorescence to excess the threshold of RT-QuIC reactions (11% or 30,000 rfu). For negative reactions that did not reach threshold during the assay, lag phase was assigned as 60 h. Shown were individual time points with error bars of means ± S.E. plotted against log-dilution series. **c** Protein aggregation rate (PAR) of RT-QuIC reactions from individual dilutions of PD, DLB, and control samples. Lag phase data (h) in **b** were converted to PAR (1/h). Shown were average rate values and error bars of S.E. Semi-log linear regression lines were applied to the PD (r^2^ = 0.91) and DLB (r^2^ = 0.88) groups. For negative reactions (assigned with a lag phase of 60 h), the rate was set at 0 (dotted line). **p < 0.01, ***p < 0.005, ****p < 0.001

To verify that our streamlined RT-QuIC assay is able to detect the seeding activity of αSyn^D^, we first examined brain homogenate (BH) prepared from neuropathologically confirmed cases of PD (n = 3), DLB (n = 3), and from NS controls (n = 3). The RT-QuIC seeding activity of αSyn^D^ aggregates in biospecimens was measured as time-dependent increases in ThT fluorescence intensity above background threshold [14, 15]. As expected, αSyn^D^ seeding activity was readily detected using our streamlined RT-QuIC assay. RT-QuIC reactions seeded by 2 µl of BH from cases of PD and DLB serially diluted to 10^–3^ through 10^–8^ showed varying degrees of ThT responses within 60 h (Fig. 1a). In contrast, no seeding activity was observed with BH from NS controls at dilutions of 10^–3^ through 10^–6^ (Fig. 1a). RT-QuIC reactions seeded by BH showed a lag phase ranging from 10 to 50 h, with DLB cases displaying shorter lag phase than PD cases at respective dilutions (Fig. 1b). To better characterize the RT-QuIC kinetics, protein aggregation rate (PAR), defined as the inverse of the lag phase (1/h), was used for measuring the rate of seeding activity at each dilution [27]. A dose-dependent increase in PAR was observed in BH-seeded RT-QuIC reactions from both PD and DLB cases (Fig. 1c). Moreover, PAR was significantly higher in DLB than PD across several orders of magnitudes in BH dilutions (Fig. 1c), consistent with higher levels of αSyn^D^ aggregates known to be present in brains of DLB patients as compared to those with PD [28]. Our results are in agreement with those in the published study of the second-generation αSyn RT-QuIC assay using in-house generated rec-Syn preparations [15], with similar RT-QuIC kinetics (within 60 h) at comparable dilutions of BH from PD and DLB. Taken together, these results confirmed the feasibility of using commercially available rec-Syn for an ultrasensitive RT-QuIC assay of αSyn^D^ in million-fold diluted BH of PD and DLB.

### Ultrasensitive detection of αSyn^D^ seeding activity in CSF samples of PD and DLB

**Fig. 2:**
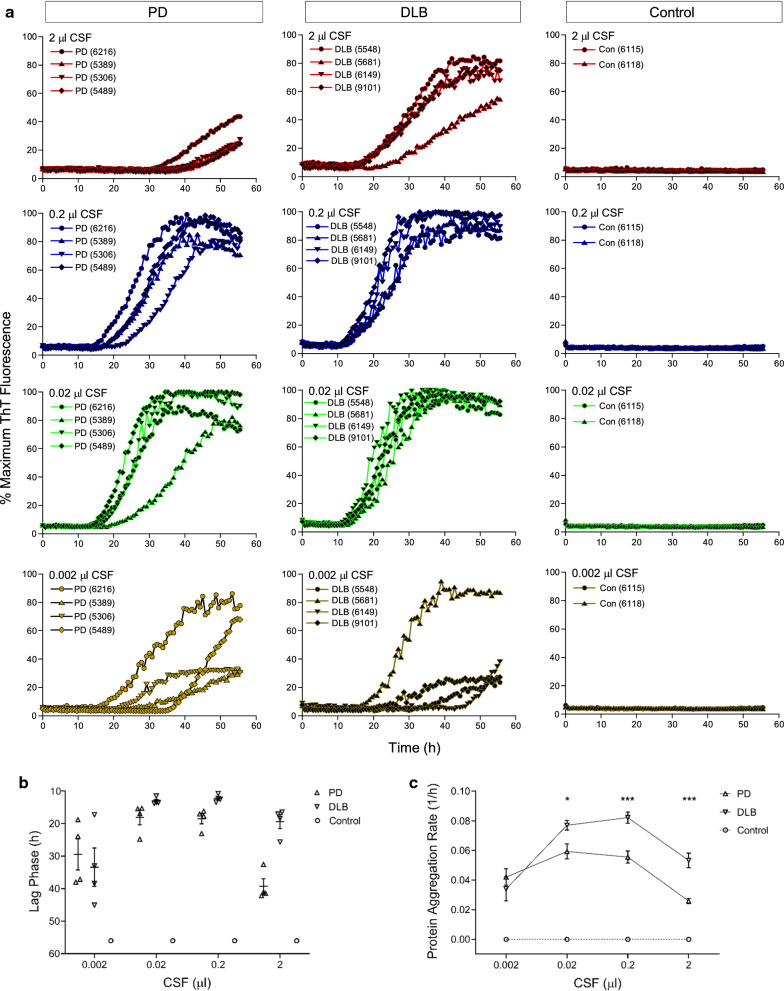
Ultrasensitive αSyn RT-QuIC assay of CSF samples of PD and DLB. **a** αSyn RT-QuIC spectra of postmortem CSF from neuropathologically confirmed cases of PD (n = 4, left panels), DLB (n = 4, middle panels), and NS controls (n = 2, right panels). Case numbers were indicated in parentheses. RT-QuIC reactions were seeded with 2 µl of CSF either undiluted or serially diluted to 10^–1^ through 10^–3^ (v/v), equivalent to 2–0.002 µl of original CSF. Average RT-QuIC reactivity was shown for individual CSF titrations in 4 cases of PD, 4 cases of DLB, and 2 cases of NS controls tested in quadruplicate. Data were expressed as percentages of the maximum ThT fluorescence. **b** Lag phase of RT-QuIC reactions seeded with individual CSF levels in PD, DLB, and control samples. RT-QuIC spectra for individual CSF levels of each case in **a** were used to obtain the time required for the average fluorescence to excess the threshold of RT-QuIC reactions (11% or 30,000 rfu). Shown were individual time points with error bars of means ± S.E. plotted against CSF levels. **c** Protein aggregation rate of RT-QuIC reactions seeded with individual CSF levels in PD, DLB, and control samples. Lag phase data (h) in **b** were converted to protein aggregation rate (1/h). Shown were average rate values and error bars of S.E. For negative reactions, the rate was set at 0 (dotted line). *p < 0.05, ***p < 0.005

After we successfully detected αSyn^D^ in highly diluted BH of PD and DLB cases, we next evaluated the sensitivity of our RT-QuIC assay for postmortem CSF samples. As reported previously, addition of a small amount of SDS (0.0015%) is necessary to bring the CSF-seeded RT-QuIC reactions to completion within 60 h [15]. In our protocol, the level of SDS was further reduced to 0.0005% (5 ppm) (see Methods section). CSF samples were acquired from neuropathologically confirmed cases of PD (n = 4), DLB (n = 4), as well as NS controls (n = 2), and 2 µl of CSF either undiluted or serially diluted to 10^–1^-10^–3^, equivalent to 2–0.002 µl of CSF, respectively, was used to seed RT-QuIC reactions. Seeding activity was detectable in PD and DLB cases but not in controls at all CSF levels, and remarkably, even in as little as 0.002 µl of CSF (Fig. 2a). The lag phase varied from 12 to 45 h in CSF-seeded RT-QuIC reactions, with a shorter lag phase in DLB than PD in RT-QuIC reactions seeded by 0.02–2 µl CSF (Fig. 2b). Accordingly, PAR was significantly higher in DLB than in PD at the respective CSF levels (Fig. 2c). Interestingly, for both PD and DLB, PAR increased when seeding went from 0.002 µl to 0.02 µl of CSF, but plateaued at 0.2 µl CSF and even decreased at 2 µl of undiluted neat CSF (Fig. 2b). Thus, non-linear RT-QuIC responses seemed to occur at higher seeding doses of CSF. This is likely due to the presence of putative inhibitors in undiluted samples that may suppress initial RT-QuIC reactions which subsequently rebound upon dilutions, consistent with the previous observations as reported [29, 30]. In summary, our streamlined RT-QuIC assay enabled ultrasensitive detection of αSyn^D^ in PD and DLB cases using only nanoliters of CSF.

### Analytical performance of the streamlined RT-QuIC assay for CSF samples of PD and DLB

**Fig. 3:**
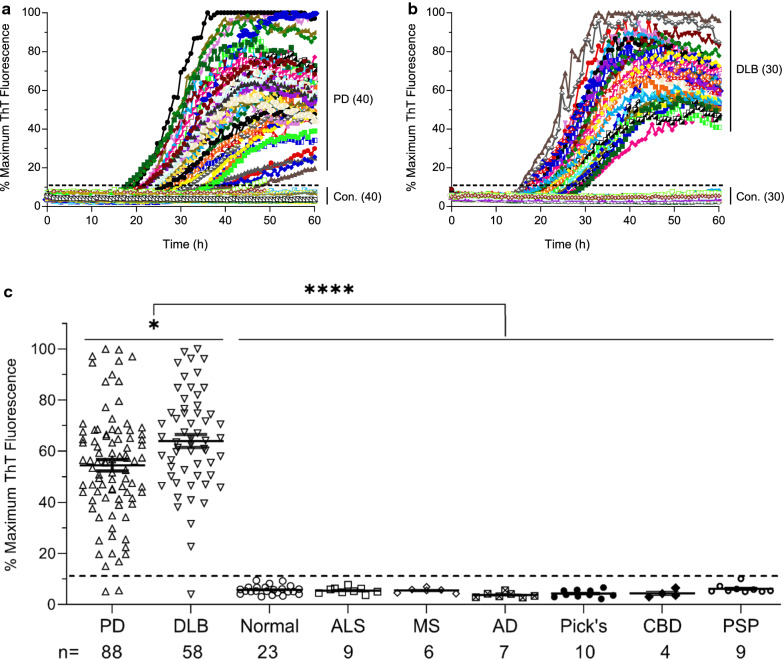
Analytical performance of αSyn RT-QuIC assay for a large collection of CSF samples from neuropathologically confirmed cases of PD, DLB, and NS controls. **a** RT-QuIC spectra of postmortem CSF samples from 40 cases of neuropathologically confirmed PD patients and 40 NS controls. **b** RT-QuIC spectra of postmortem CSF from 30 cases of DLB patients and 30 NS controls. **c** Scattered plot of RT-QuIC reactivity as percentages of maximum ThT fluorescence at the end of 60 h assay of 214 neuropathologically confirmed CSF cases, including those with PD (n = 88) and DLB (n = 58), as well as NS control cases (n = 68) including those neurologically normal (n = 23), and those with amyotrophic lateral sclerosis (ALS, n = 9), multiple sclerosis (MS, n = 6), Alzheimer’s disease (AD, n = 7), Pick’s disease (n = 10), corticobasal degeneration (CBD, n = 4), and progressive supranuclear palsy (PSP, n = 9). Horizontal bars were means ± S.E. of ThT fluorescence for each group of CSF cases. The dotted line represents the threshold (11%) defining the positive and negative cases. All RT-QuIC reactions were seeded with 200 nl of CSF (2 µl of tenfold-diluted CSF) in quadruplicate wells per sample. *p < 0.05 comparing PD with DLB, **** p < 0.0001 comparing the combined PD and DLB group with all of the NS controls

To further evaluate the diagnostic utility of the streamlined αSyn RT-QuIC assay, we acquired a large number of postmortem CSF samples from neuropathologically confirmed cases of PD, DLB, and NS controls from the NIH NeuroBioBank (NBB), a centralized network of biorepositories. All CSF samples were evaluated at the 0.2 µl seeding level (2 µl of tenfold dilution of neat CSF) for RT-QuIC reactions in quadruplicate with a throughput of 24 samples (including positive and negative controls) in a single 96-well plate per assay. We first examined the RT-QuIC profile in a set of 40 cases of PD and 40 NS controls. CSF samples from PD cases displayed positive RT-QuIC responses within ~ 18–50 h (Fig. 3a). For comparison, we tested another set of 30 cases each for DLB and NS controls. RT-QuIC of CSF samples from DLB cases exhibited strong ThT responses within ~ 15–30 h (Fig. 3b). NS controls showed negative reactivity below the threshold within 60 h. The overall RT-QuIC kinetics of CSF samples from PD cases exhibited a slower and broader RT-QuIC reactivity as compared to that of DLB (Fig. 3a vs. Figure 3b), as observed in a previous study of a small cohort of CSF samples from PD and DLB patients [15], suggesting a differential pattern of RT-QuIC kinetics between CSF samples from PD and DLB patients.

In total, our RT-QuIC assay examined 214 postmortem CSF samples from 88 cases of PD, 58 cases of DLB, and 68 cases of NS controls [neurologically normal (n = 23), ALS (n = 9), MS (n = 6), AD (n = 7), Pick’s disease (n = 10), CBD (n = 4), and PSP (n = 9)]. Analysis of the CSF seeding activity at the end of the 60 h RT-QuIC assay revealed an excellent analytical performance for both PD and DLB (Fig. 3c). Positive seeding activity was observed in 86 out of 88 CSF samples in the PD group and 57 out of 58 CSF samples in the DLB group, yielding a sensitivity of 98% for both PD and DLB. None of the 68 NS control CSF samples showed positive seeding activity, resulting in a specificity of 100%. In comparison, the overall CSF seeding activity was significantly higher in DLB than PD (*p* < 0.05), and the combined PD and DLB group was well above NS controls (*p* < 0.0001). Taken together, these results confirm that CSF is a useful specimen for validating the RT-QuIC assay as a robust tool for the diagnosis of PD and DLB.

### Single RT-QuIC assay platform for multiple biospecimens of PD

**Fig. 4:**
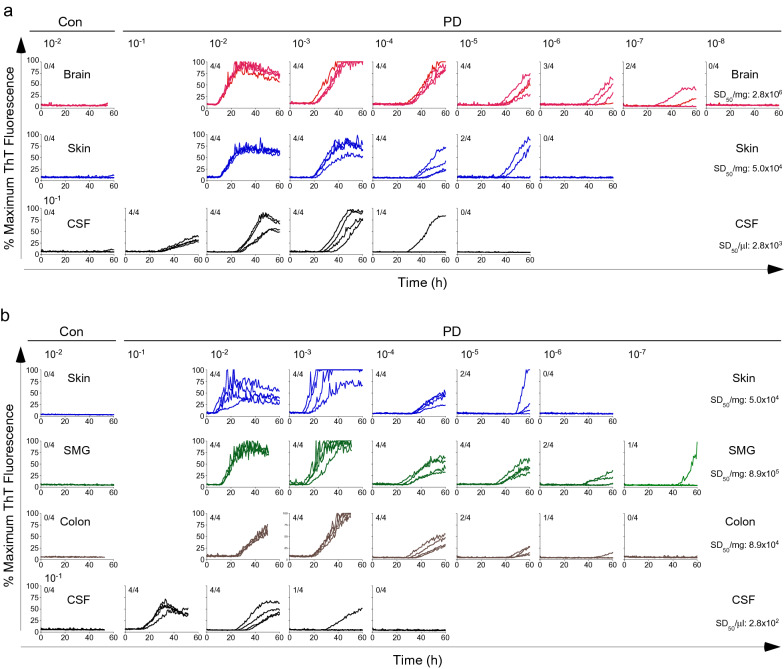
Comparative analyses of αSyn^D^ seeding activity in multiple biospecimens of PD by end-point dilution RT-QuIC assay. **a** RT-QuIC spectra of a neuropathologically confirmed case of PD and a NS control affected by AD (Con) using serially diluted homogenates of the brain and scalp skin, as well as CSF. **b** RT-QuIC spectra of a neuropathologically confirmed case of PD and a NS control without any neurological disease (Con) using serially diluted homogenates of scalp skin, SMG, and sigmoid colon, as well as CSF. The tenfold serially dilutions were indicated on the top of **a** and **b,** and 2 µl from the designated dilutions was used to titrate RT-QuIC reactivity to background levels within 50–60 h. Shown were ThT fluorescence traces from 4 replicate reactions at the designated dilutions. The fractions in the upper left corner of each graph indicated the ThT-positive/total replicate reactions. A threshold of 11% was used to define the positive and negative reactivity. SD_50_ values (per mg or µl) derived from Spearman-Kärber analyses of end-point dilution results for the PD cases were shown for each type of biospecimens on the right

Finally, we examined whether our streamlined RT-QuIC assay can be used for different types of specimens without the need to modify assay conditions. Several types of biospecimens were collected from the same PD cadavers, subjected to the same processing and serial dilution procedure, and tested under the same RT-QuIC assay conditions. Spearman-Kärber endpoint dilution analysis was used to determine the seeding dose at which 50% of replicate reactions were positive, termed SD_50_ [24]. In a neuropathologically confirmed PD case from whom scalp skin was available in addition to the brain and CSF, we performed parallel RT-QuIC reactions seeded with the respective specimens in tenfold dilution series (from 10^–1^ through 10^–8^). As shown in Fig. 4a, αSyn^D^ seeding activity displayed dose-dependent titration in serially diluted PD samples of BH, skin homogenate, and CSF, respectively. The Spearman-Kärber analysis of this PD case yielded SD_50_ values at 2.8 × 10^6^/mg for the brain, 5.0 × 10^4^/mg for the skin, and 2.8 × 10^3^/µl for CSF. No seeding activity was detected in corresponding biospecimens from a control cadaver affected by AD (Fig. 4a). To explore more broadly the tissue distribution of αSyn^D^, another case of neuropathologically confirmed PD was examined from which scalp skin, submandibular salivary gland (SMG), and sigmoid colon were available in addition to CSF. Using the same RT-QuIC assay protocol, αSyn^D^ seeding activity was examined in serially diluted tissue homogenates of skin, SMG, and colon, and in serially diluted CSF (Fig. 4b). SD_50_ values obtained from Spearman-Kärber analysis of this PD case were 5.0 × 10^4^/mg for skin, 8.9 × 10^5^/mg for SMG, 8.9 × 10^4^/mg for colon, and 2.8 × 10^2^/µl for CSF. In contrast, no seeding activity was found in corresponding specimens from a neurologically normal control case (Fig. 4b). Therefore, the αSyn^D^ seeding activity in PD peripheral tissues of the skin, SMG, and colon was relatively high, ranging from ~ 10^4^ to ~ 10^5^ per mg of tissues, which was only 1 to 2 orders of magnitude lower than that in the brain (~ 10^6^/mg). The seeding activity in PD CSF was in the range of 10^2^–10^3^ per µl. For brain and CSF, the previously reported SD_50_ values in PD and DLB were in the range of 10^5^–10^6^/mg and 4–54/µl, respectively [15]. Thus, our streamlined RT-QuIC assay provides a single unified protocol for robust tracking of αSyn^D^ seeding activity across multiple biospecimens from the same patients affected by PD and other synucleinopathies.

**Fig. 5:**
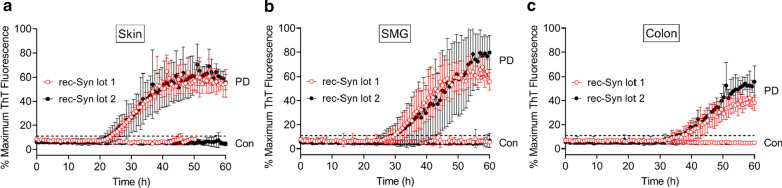
Reliability of RT-QuIC assay for PD biospecimens using different batches of rec-Syn substrate. **a** RT-QuIC spectra of scalp skin homogenate from a case of neuropathologically confirmed PD or a NS control without any neurological disease using two different batches of rec-Syn substrate (lot 1 and lot 2). Individual traces were means ± SD of ThT fluorescence from the PD skin sample (PD) assayed with rec-Syn lot 1 and lot 2 (n = 6 for both lots) or the control skin sample (Con) assayed with rec-Syn lot 1 (n = 3) and lot 2 (n = 6). **b** RT-QuIC spectra of SMG homogenate from the same PD and control cases as in **a** using rec-Syn lot 1 and lot 2. Individual traces were means ± SD of ThT fluorescence from the PD SMG sample (PD) assayed with rec-Syn lot 1 (n = 6) and lot 2 (n = 4) or the control SMG sample (Con) assayed with rec-Syn lot 1 and lot 2 (n = 6 for both lots). **c** RT-QuIC spectra of sigmoid colon homogenate from the same PD and control cases as in **a** using rec-Syn lot 1 and lot 2. Individual traces were means ± SD of ThT fluorescence from the PD colon sample (PD) assayed with rec-Syn lot 1 and lot 2 (n = 4 for both lots) or the control colon sample (Con) assayed with rec-Syn lot 1 (n = 5) and lot 2 (n = 4). The dotted line represents the threshold of positivity (11%). The specific batches of rec-Syn substrate used were lot 1 (082517AS) and lot 2 (111317AS) from rPeptide. RT-QuIC assay of skin, SMG, and sigmoid colon specimens was performed using the same protocol as described in Methods, in which 2 µl of tissue homogenates diluted to 10^–3^ (w/v) was used to seed each reaction

To evaluate the reliability of our streamlined RT-QuIC protocol, we analyzed tissue homogenates from a third case of neuropathologically confirmed PD using different batches of rec-Syn substrate. Distinct patterns of RT-QuIC kinetics from reactions seeded by PD tissue homogenates from the skin, SMG, and colon were consistently reproduced when performed with two different lots of rec-Syn (Fig. 5). In contrast, only negative reactivity was observed in corresponding tissue homogenates from a neurologically normal control case, confirming the specificity of the RT-QuIC reactions (Fig. 5). Therefore, our streamlined RT-QuIC assay enables reliable and robust analysis of αSyn^D^ seeding activity in various patient-derived specimens.

## Discussion

αSyn RT-QuIC has recently gained popularity due to its ability to detect the seeding activity of αSyn^D^ aggregates in clinical specimens. Initial applications have been demonstrated by several groups using BH and CSF of PD and DLB. However, existing αSyn RT-QuIC assay protocols vary in configuration, length of assay, tissue processing, as well as the compositions and sources of reagents [14–17, 31]. We have now developed a streamlined αSyn RT-QuIC assay for various biospecimens of PD and DLB, with optimized reagent compositions and a single tissue extraction procedure, resulting in a simplified and improved assay platform that can be easily implemented in a routine laboratory.

A key reagent for the αSyn RT-QuIC assay is rec-Syn that serves as the substrate for seeded fibrillization. In many published studies, rec-Syn is produced in-house, which often introduces batch-to-batch variability and requires considerable expertise and personnel to ensure the production of high quality rec-Syn protein during prolonged in-house preparation and purification steps (~ 5–7 days). We have developed a streamlined αSyn RT-QuIC assay based on the second-generation platform [15] and opted for entirely commercial reagents including rec-Syn in an effort to simplify and standardize the assay conditions. As a result, our streamlined αSyn RT-QuIC assay requires a single technician with a hands-on time of ~ 1 h to set up 96-reaction wells in a multi-well plate for unattended readouts from a plate reader. Therefore, our simplified protocol drastically lessens the technical barrier to the implementation of the highly efficient αSyn RT-QuIC assay in a routine laboratory. An upward adjustment of ThT concentrations (from 10 µM to 20 µM) was also incorporated to offset the self-quenching of ThT fluorescence upon binding to amyloid fibrils as reported in a previous study [26].

We have extensively tested our streamlined αSyn RT-QuIC protocol against neuropathologically confirmed cases of PD and DLB using BH and CSF. To validate its diagnostic value for PD and DLB, we performed αSyn RT-QuIC analysis of a large panel of 214 CSF samples, yielding an overall sensitivity of 98% and a specificity of 100%. Our results are consistent with other reports of overall high diagnostic accuracy of αSyn RT-QuIC analyses of CSF from various PD and DLB cohorts from different populations [14–17, 31]. Therefore, it is foreseeable that a standardized RT-QuIC assay of CSF will offer added diagnostic values for both PD and DLB. Notably, we observed αSyn^D^ seeding activity in a much lower sample volume (0.02–0.2 µl) of ventricular CSF obtained at autopsy than that required for premortem lumbar CSF (15–20 µl) [14–17, 31]. This discrepancy could be due to higher levels of αSyn^D^ in autopsy CSF than those in premortem patients. Alternatively, the assay sensitivity could be influenced by a difference in biochemical and cytological compositions between ventricular and lumbar CSF [32], including a difference in the levels of salts [33] since some inorganic ions have been found to modulate the performance of RT-QuIC in different sample matrices [34]. Nonetheless, our findings have confirmed the value of archived CSF from well-characterized cases as a reference material for RT-QuIC assay development and validation.

Previous studies using IHC staining have observed abnormally phosphorylated αSyn in non-CNS organs of PD and DLB patients [9–11, 35–38]. However, technical challenges of the IHC-based method may have limited its clinical utility [39]. Most recently, several tissue-specific αSyn RT-QuIC platforms have been reported with nasal brushing [18], SMG [19] and skin [20, 21] samples from PD patients, but procedures for tissue extraction and reagent compositions for RT-QuIC assay differed for individual tissues and among different studies. Therefore, a simplified and standardized protocol is desired to uniformly perform RT-QuIC assay and compare distribution of αSyn^D^ among multiple tissue types. In our assay protocol, we extracted all tissues with bead-based homogenization in the same buffer, eliminated an enzymatic digestion step lasting for 4 h, and performed RT-QuIC assay under identical conditions. Thus, we have provided a single and simplified RT-QuIC protocol for concurrent detection of αSyn^D^ in different tissues. Using Spearman-Kärber analysis of endpoint dilution experiments, we have demonstrated the utility of our RT-QuIC assay for a parallel and quantitative comparison of αSyn^D^ seeding activity (in SD_50_ units) for multiple PD specimens including the skin, SMG, colon, and CSF, all of which are clinically accessible. In short, our RT-QuIC assay protocol supports the measurement of αSyn^D^ seeding activity in different biospecimens under the same assay conditions.

Our RT-QuIC assay has enabled ultrasensitive detection of αSyn^D^ seeding activity in highly diluted specimens of PD and DLB, up to million-fold dilution for BH and several thousand-fold dilution for peripheral tissues, and in nanoliters of CSF. Given the high costs of patient recruitment and specimen procurement, the extremely low sample consumption afforded by our RT-QuIC assay will facilitate the efficient use and sharing of scarce resources of biospecimens collected during various clinical studies. As a reliable and robust tool, the ultrasensitive RT-QuIC assay platform has several attributes that will allow for easy translation into the clinical arena, including rapid turnaround, high throughput, and ultrasensitive and automated readouts. Indeed, the prion RT-QuIC assay of CSF has been incorporated into the updated diagnostic criteria for Creutzfeldt-Jakob disease since 2018 [40]. Future efforts to incorporate the αSyn RT-QuIC assay into clinical practice will require analytical and clinical validation using large collections of premortem patient samples at multiple sites. It is plausible that a standardized and clinically validated αSyn RT-QuIC assay for clinically accessible biospecimens, including CSF and various peripheral tissues, will serve as a diagnostic biomarker of PD and DLB.

## Conclusions

In summary, we have developed a streamlined RT-QuIC assay platform for ultrasensitive detection of αSyn^D^ in a diverse array of biospecimens from postmortem PD and DLB patients. The streamlined protocol requires minimal hands-on time and technical expertise to set up the RT-QuIC assay. This optimized platform could facilitate easy implementation of this αSyn RT-QuIC assay in a routine laboratory, and enable the standardization of the assay across different sites. We have verified the analytical sensitivity and specificity of a streamlined RT-QuIC assay in a large number of postmortem CSF samples of PD and DLB cases. Moreover, we have demonstrated that a single RT-QuIC protocol is suitable to detect αSyn^D^ seeding activity in multiple tissue specimens such as the brain, skin, salivary gland, and colon. Finally, our assay is ultrasensitive, high-throughput, low on sample consumption, and easy to adapt to different biospecimens. The ongoing evaluation of αSyn^D^ as a potential biomarker of PD and DLB in clinically accessible biospecimens has broad implications for understanding disease pathogenesis, improving clinical and differential diagnosis, and monitoring efficacy of treatments and neuroprotective agents in clinical trials.

## Data Availability

All data are available upon reasonable request to the corresponding authors.
